# Grafting Thin Layered Graphene Oxide onto the Surface of Nonwoven/PVDF-PAA Composite Membrane for Efficient Dye and Macromolecule Separations

**DOI:** 10.3390/nano10040792

**Published:** 2020-04-20

**Authors:** Febri Baskoro, Selvaraj Rajesh Kumar, Shingjiang Jessie Lue

**Affiliations:** 1 Department of Chemical and Materials Engineering, Chang Gung University, Guishan District, Taoyuan City 333, Taiwan; febri_baskoro@yahoo.co.id (F.B.); rajeshkumarnst@gmail.com (S.R.K.); 2 Department of Safety, Health and Environmental Engineering, Ming Chi University of Technology, Taishan District, New Taipei City 243, Taiwan; 3 Department of Orthopedic Surgery, Chang Gung Memorial Hospital, Anle District, Keelung City 204, Taiwan

**Keywords:** graphene oxide, composite membrane, permeance, dye rejections, antifouling effects

## Abstract

This study investigates the permeance and rejection efficiencies of different dyes (Rhodamine B and methyl orange), folic acid and a protein (bovine serum albumin) using graphene oxide composite membrane. The ultrathin separation layer of graphene oxide (thickness of 380 nm) was successfully deposited onto porous polyvinylidene fluoride-polyacrylic acid intermediate layer on nonwoven support layer using vacuum filtration. The graphene oxide addition in the composite membrane caused an increased hydrophilicity and negative surface charge than those of the membrane without graphene oxide. In the filtration process using a graphene oxide composite membrane, the permeance values of pure water, dyes, folic acid and bovine serum albumin molecules were more severely decreased (by two orders of magnitude) than those of the nonwoven/polyvinylidene fluoride-polyacrylic acid composite membrane. However, the rejection efficiency of the graphene oxide composite was significantly improved in cationic Rhodamine B (from 9% to 80.3%) and anionic methyl orange (from 28.3% to 86.6%) feed solutions. The folic acid and bovine serum albumin were nearly completely rejected from solutions using either nonwoven/polyvinylidene fluoride-polyacrylic acid or nonwoven/polyvinylidene fluoride-polyacrylic acid/graphene oxide composite membrane, but the latter possessed anti-fouling property against the protein molecules. The separation mechanism in nonwoven/polyvinylidene fluoride-polyacrylic acid membrane includes the Donnan exclusion effect (for smaller-than-pore-size solutes) and sieving mechanism (for larger solutes). The sieving mechanism governs the filtration behavior in the nonwoven/polyvinylidene fluoride-polyacrylic acid/graphene oxide composite membrane.

## 1. Introduction

Throughout the world, rapid industrial development from photo-electrochemical cells, papers, plastics, textiles, pharmaceuticals, etc. has brought about serious water contamination. Thereby, wastewater treatment has emerged to solve the water crisis for fast population growth [1,2]. Based on the evaluation of the World Health Organization (WHO), 1.1 billion people could face a shortage of clean drinking water. According to World Water Council reports, the ‘water scarce’ population will increase to 3.9 billion people by 2030 [3]. In this context, several researchers are focusing on salt/dye/protein/oil-effluents water treatment, targeting the emission standard of wastewater [2]. Various scientific techniques including adsorption, ion exchange, chemical oxidation, flocculation and photocatalytic degradation are established to separate contaminated effluents from wastewater [4,5]. Based on these methods, many technical reports were established for the possible treatment and processing of drinking water. However, these conventional processes generate secondary waste, use additional chemicals, or require large footprint handling sites. Therefore, the development of novel technology to remove the contamination of salts, dyes or proteins from wastewater is essential in both theoretical and practical studies [6].

Currently, polymeric membrane technology growth (which subsidizes up to 53% of total world processes for clean water production [3]) is employed to process wastewater treatments. Among the many polymers, the most widely used membrane separation synthetic polymers are polyvinylidene fluoride (PVDF). PVDF is a semi-crystalline polymer with facile film-forming properties that has good mechanical strength, high chemical and oxidation resistance, thermal stability and membrane flexibility [7]. Nevertheless, the pristine PVDF membrane has lower water permeation and separation efficiencies owing to its high hydrophobicity and poor anti-fouling resistances, which limits the practical application of various contaminations [8]. To overcome the above drawbacks, various approaches to surface modification have been employed to enhance the hydrophilicity, selectivity and antifouling properties of the PVDF membrane. Numerous surface modifications, including polytetrafluoroethylene [9], polydimethylsiloxane [10], polyethylene glycol [11,12], polyvinyl alcohol [13], polyacrylic acid (PAA) [14], polyvinyl pyrrolidone [15], etc., were implemented to improve the surface hydrophilicity, porosity and permeate flux [16]. Among these polymers, polyacrylic acid (PAA) coatings using layer-by-layer assembly have been considered as a marginal surface modification owing to its simplicity, versatility and non-toxicity. The addition of PAA with a PVDF membrane significantly changes the structural, surface and micro-porous properties [14]. Gonzales et al. [17] reported that the PAA monomer chain containing a carboxylic acid group improves the PVDF-PAA negative surface charge and hydrophilicity of the composite membrane. Chen et al. [18] and Natalie et al. [19] reported that the presence of PAA in PVDF composite membrane enhances the hydrophilic nature which dramatically improves the membrane performance in both water permeation flux and rejection rates. Therefore, a PVDF membrane with PAA could pointedly improve the water contact angle and surface charges that contribute to higher water permeation, separation efficiency and anti-fouling resistance compared with the pristine PVDF membrane [20,21,22]. However, the weak physical/chemical barrier effects on the PVDF-PAA membrane in various pH conditions limit the long-term stability and its usage for commercial purposes [23].

In recent years, the blending or coating of various nanomaterials such as Ag, TiO_2_, SiO_2_, Al_2_O_3_, Fe_3_O_4_, ZrO_2_, carbon nanotubes and graphene/graphene oxide (GO) into the polymeric matrix [5,24,25,26,27,28,29] has gained much attention for making effective filtration membranes. Among many nanomaterials, there has been extensive fascination with two-dimensional ultrathin GO has for its use as micro/ultrafiltration membrane due to its superior chemical resistance, mechanical strength, thermal and surface properties [30,31]. Experimental and theoretical examinations confirmed that graphene based composite membranes have excellent nanochannels for permeation and high rejection of molecules/ions with long-term stability [32]. The distribution or alignment of GO in the PVDF polymeric matric could depend on various experimental techniques including electrospinning [33], direct blending method [25], immersion phase inversion [34], coagulation and immersed precipitation method [35], etc. The GO distribution in the polymeric matrix could improve the composite membrane properties including porosity, viscosity, surface charge, mechanical strength, contact angle and antifouling efficiency, respectively [36]. When GO was blended into a PVDF matrix, the resulting composite membranes have a three times higher water flux than that of pure PVDF membranes due to their controlled pore sizes and surface properties [37]. Similarly, Ghaffar et al. reported that an electrospun PVDF/GO nanofibrous membrane improved the selectivity (99%) toward positively charged dyes based on electrostatic attraction while maintaining rejection (100%) for negatively charged dye than that of the pure PVDF membrane [33]. Recently, Chen et al. used a PVDF/polyethylene glycol composite for GO functionalization to improve hydrophilicity, surface charge and pore size that enhanced the permeability and antifouling properties [38].

In addition, the GO-rich separation top layer is fabricated onto a porous or dense support using a vacuum filtration method [20], spin-coating or dip coating method [39] without the use of binder. The electrostatic interaction between cations and GO resulted in various interlayer spacing values (2.8–6.1 nm) and net charges, which affect the permeance flux and ion rejections. This GO layer improved salt rejections by 2–5 times compared with the GO-free control membrane [20].

In this work, the composite consisting GO top layer on nonwoven/PVDF-PAA (i.e., three-component) [20] and the control membrane without GO layer, nonwoven/PVDF-PAA (two-component) membrane, are investigated for the permeance and rejection values of several model molecules. These include cationic Rhodamine B (Rh-B) dye, anionic methyl orange (MO), folic acid (FA) and bovine serum albumin (BSA) protein. The laminar GO separation layer is more hydrophilic, with a tighter pore structure and much lower surface charge than the control nonwoven/PVDF-PAA membrane. The filtration behaviors of these model molecules through the GO composite and the control membrane are elucidated.

## 2. Materials and Methods

### 2.1. Synthesis of Graphene Oxide (GO)

Modified Hummer’s routine was used to synthesis the GO nanosheets [20,40]. Initially, 2 g graphite flakes (Sigma-Aldrich, St. Louis, MO, USA) were dissolved in 300 mL of sulfuric acid (H_2_SO_4_, 98%, Scharlab S.L., Barcelona, Spain) at ambient temperature and stirred well for 15 min. Then, 2 g potassium permanganate (KMnO_4_, Nihon Shiyaku Industries Ltd., Osaka, Japan) was mixed with above solution with continuous stirring. A subsequent amount of KMnO_4_ was added slowly when the green MnO^3−^ color diminished and total of 10 g KMnO_4_ was added. An appropriate amount of ice cubes was mixed into the solution once completing the reaction. Afterwards, the chemical reactions of graphene exfoliation process were performed in an ice bath, resulting in the solution exposing a purple color. After being leftover overnight, the precipitate was isolated from the solution and dissolved in 100 mL deionized (DI) water. The precipitate was washed with DI water many times until the pH became neutral. The precipitate GO particles were separated using centrifugation and dried at 60 °C under vacuum oven. The dried sample was again dispersed in DI water to form GO solution.

### 2.2. Synthesis of Nonwoven/PVDF-PAA Composite

The thermoplastic polyethylene terephthalate (PET, Ahlstrom filtration Co., Helsinki, Finland) was used to prepare a nonwoven support to increase the composite mechanical strength. An intermediate layer of polyvinylidene fluoride-polyacrylic acid (PVDF-PAA, Sigma-Aldrich, St. Louis, MO, USA) was cast on the nonwoven from 20 wt% PVDF and 2 wt% PAA in N-methyl-2-pyrrolidinone (NMP, Mallinckrodt Baker Inc., Philipsburg, PA, USA) solution. The PAA additive provides hydrophilicity and compatibility with GO top layer. The casted polymeric film was place in a coagulation bath (DI water) for the phase inversion taking place and the membrane dried at 60 °C. The dried nonwoven/PVDF-PAA sample was also denoted as two-component composite membrane.

### 2.3. Synthesis of Nonwoven/PVDF-PAA/GO Composite Membrane

**Figure 1:**
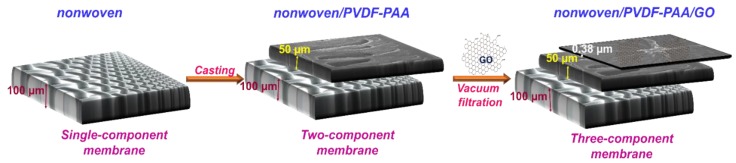
Schematic diagram for the formation of three-component composite membrane.

The nonwoven sample was used as a support layer to improve the mechanical strength, PVDF-PAA acted as a porous intermediate layer and GO acted a separation layer in the composite membrane, as shown in Figure 1. The vacuum filtration method was used to prepare the nonwoven/PVDF-PAA/GO composite [20]. In detail, the GO solution was prepared by dissolving dried GO in DI water via an ultra-sonication. Before the GO solution was deposited onto the surface of nonwoven/PVDF-PAA membrane, it was centrifuged (1000 rpm) at ambient temperature for 1 h to remove excessive large aggregates. Then, the GO suspension was filtrated through the two-component composite membrane using vacuum filtration by applying the suction force of a water aspirator with a wetting agent (2 mL isopropyl alcohol obtained from Mallinckrodt Baker Inc., Philipsburg, PA, USA). The GO deposition rate on the composite membrane was 0.097 ± 0.013 mg cm^−2^ by filtering 10 mL of 200 ppm GO solution. The resulting membranes were dried in vacuum oven at 60 °C for overnight before filtration testing performance. The dried nonwoven/PVDF-PAA/GO sample was also denoted as three-component composite membrane.

### 2.4. Characterization

The surface morphologies of the pristine GO, nonwoven/PVDF-PAA and nonwoven/PVDF-PAA/GO composite membranes were observed using a field emission scanning electron microscope (FESEM, model JSM-7500F, Hitachi High-Technologies Corp., Tokyo, Japan). The cross-sectional microstructure of the composite membranes was observed after being freeze-fractured in liquid nitrogen. The pristine GO morphology was observed by transmission electron microscope (TEM, JEM 2000EXII, JEOL, Tokyo, Japan). The chemical structure and functional groups of graphite and pristine GO was analyzed using Fourier transform infrared spectroscopy (FT-IR, model Horiba FT-730, Minami-ku, Kyoto, Japan). Furthermore, the structural properties of graphite and pristine GO were analyzed using a micro-Raman spectroscope (Labram Hr800, Horiba, Ltd., Kyoto, Japan) at a wavelength of 785 nm. X-ray photoelectron spectroscopy (XPS K-Alpha, VG Microtech MT-500, Thermo Fisher Scientific Inc., Waltham, MA, USA) was used to examine the chemical composition and percentage of functional groups on pristine GO samples. Dynamic light scattering (Zetasizer, 2000 HAS, Malvern, Worcestershire, UK) was used to estimate the surface charges and the particle size distribution of various dyes and macromolecules. The contact angle measuring system (G10, Kruss GmbH, Hamburg, Germany) was employed to analyze the hydrophilicity of the composite membranes.

### 2.5. Filtration Analysis of Dyes And Proteins

To investigate the filtration process, the composite membrane was mounted into cross-flow filtration module with an effective filtration area of 10.75 × 10^−4^ m^2^. Constant pressure (0.49 MPa) was applied for all sample analyses at ambient temperature. The molecular weight and molecular structure of different dyes (Rh-B and MO), folic acid (FA) and protein (BSA) are listed in Table 1. The feed volume was 1 L DI water containing feed solutions of 10 ppm dye solutions (typical dye concentration in synthetic dye wastewater or treated real textile effluent [41,42]) or 500 ppm of FA or BSA solutions (typical concentration in fermented vegetable solution containing FA [43] and BSA composition in whey [44]). The permeance values at steady state were calculated and continuously analyzed at 20-min intervals. The rejection efficiencies of ionic solutions (dyes and FA) and BSA protein were measured using UV-Visible spectrophotometer (V-650, Jasco, Hachioji, Tokyo, Japan). The Rh-B and MO dyes were analyzed at a wavelength of 554 nm and 466 nm, respectively. For FA and BSA molecules, the solution adsorbance at 281 nm and 280 nm were measured, respectively. The water flux, permeance and the rejections were calculated using the Equations (1)–(3), respectively [20].

(1) $$J=\frac{m}{A.h}$$

(2) $$P=\frac{m}{A.h.\Delta P}$$

(3) $$R\;\left(\%\right)=\left(1-\frac{C_{2}}{C_{1}}\right)\times 100\;$$

where *A* is the filtration area (m^2^), h is the period of time (h), *J* is the water flux (kg m^−2^ h^−1^), ∆P is the operating pressure condition (MPa), P is the permeance through the filtration membrane (kgm^−2^ h^−1^ Mpa^−1^), m is the weight of the permeate solution (kg), *C*_1_ is concentration of the feed solution and *C*_2_ is concentration of the permeate and R is the rejection efficiency (%). In order to acquire reliable data, all the sample performances were repeated three times (n = 3) and the average values were reported.

## 3. Results

### 3.1. Morphological and Structural Properties of GO

**Figure 2:**
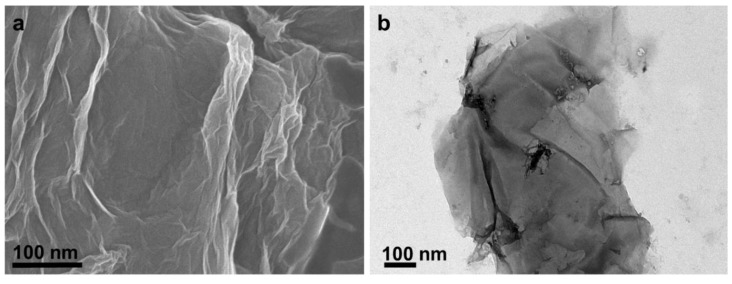
(**a**) Field emission scanning electron microscope (FESEM) and (**b**) transmission electron microscopic (TEM) images of synthesized graphene oxide (GO).

The surface micrograph of the GO was analyzed using FESEM, as shown in Figure 2a. The FESEM image revealed GO as wrinkled-sheet like structure with the size ranged 0.1–1.5 µm. Moreover, this wavy wrinkled shape and single-layered GO nanosheet was clearly observed in TEM image, as shown in Figure 2b. FTIR spectroscopy was used to investigate the chemical structure of the graphite and GO, as shown in supplementary information (Figure S1). No significant oxygen-containing functional groups were observed in the graphite whereas many characteristic peaks were observed in the GO nanosheets. The small FTIR peaks at 1059 cm^−1^ and 1225 cm^−1^ denoted the C-O stretching vibration of epoxy alkyl group. The sharp peak at 1723 cm^−1^ signified the C=O stretching vibration of GO. The adsorbed hydroxyl molecules and vibration of aromatic C=C bond was observed at 1621 cm^−1^. Two characteristic FTIR peaks at 3368 cm^−1^ and 1370 cm^−1^ were corresponding to the stretching vibration and deformation vibration of hydroxyl groups present in GO. All the corresponding FTIR peaks confirmed the GO formation via graphite exfoliation [20,45]. Furthermore, Raman spectroscopy was employed to investigate the chemical structure and I_D_/I_G_ ratio of graphite and GO nanosheets (Figure S2). The intense peak at 1590 cm^−1^ (G-band) corresponds to distinct sp^2^ carbon structure due to first order E_2g_ scattering modes [46]. The significant Raman peak of GO at 1354 cm^−1^ (D-band) confirmed the oxidation in graphite owing to structural changes. The intensity ratio of D band over G band of graphite was 0.15 whereas the ratio increased to 1.03 for GO sample. Compared with original graphite (~2720 cm^−1^), the 2D peak of GO was slightly shifted to ~2672 cm^−1^ and its peak intensity was significantly suppressed. This indicating the formation of a single layer of GO nanosheet [20].

**Figure 3:**
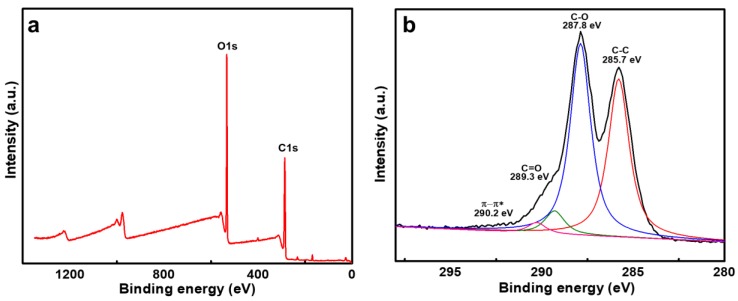
(**a**) full scan and (**b**) detailed C1s scan of GO using X-ray photoelectron spectroscopy.

The GO chemical composition was further investigated using XPS. The full scan XPS spectrum showed presence of 65% carbon and 35% oxygen content after the exfoliation of graphite, as shown in Figure 3a. The results confirm the higher oxidation level than the graphite (98% carbon and 2% oxygen) structure. In addition, the C1s core spectrum and its deconvolution peaks were represented in Figure 3b. The binding energies at 290.2, 289.3, 287.8 and 285.7 eV correspond to the carbon bonding of O-C=O, C=O, C-O and C-C, respectively. The higher oxidation of GO confirms the presence of more hydrophilic groups.

### 3.2. Surface and Cross-Sectional Morphologies of GO Composite Membrane

**Figure 4:**
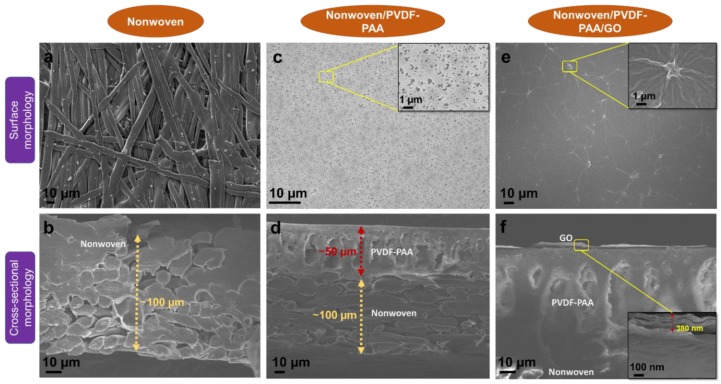
Surface and cross-sectional morphologies of (**a**,**b**) nonwoven, (**c**,**d**) nonwoven/ polyvinylidene fluoride-polyacrylic acid (PVDF-PAA) and (**e**,**f**) nonwoven/PVDF-PAA/GO composite membranes (inserted figures in (**c**,**e**,**f**) represent its higher magnifications).

The top-surface and cross-sectional morphologies of the nonwoven, nonwoven/PVDF-PAA (two-component) and nonwoven/PVDF-PAA/GO (three-component) composite membranes were observed using FESEM, as shown in Figure 4a–f. The surface morphology of the nonwoven membrane has an irregular nesting structure (Figure 4a) with an average pore size of 3–9 µm. The nonwoven thickness was about 100 µm, as observed from cross-sectional image (Figure 4b). Casting an intermediate PVDF-PAA layer onto the nonwoven surface resulted in a micro-porous structure (Figure 4c). Furthermore, the cross-sectional micrograph showed this PVDF-PAA layer had a finger-like structure with a thickness of 50 µm (Figure 4d). The pore size of the PVDF-PAA layer was reduced to 75–100 nm [20], more suitable for GO nanosheet deposition. The surface morphology of the nonwoven/PVDF-PAA/GO was changed dramatically after GO deposition. The GO layer evenly shielded the porous structure underneath with a thickness of ~380 nm (Figure 4f).

### 3.3. Surface Charge and Contact Angle Measurements

The surface charge was determined on the membranes. The porous PVDF (prepared using similar method) had a negative zeta potential of −17.3 mV in DI water [47]. The PVDF-PAA intermediate layer (with 10:1 mass ratio) showed a zeta potential of −19.4 mV, lower than that of PVDF, due to the protonation of PAA ionomer. The GO top layer demonstrated an even lower zeta potential of −30.9 mV, close to the zeta potential of the GO suspension [20,48]. The presence of carboxylate groups in GO significantly contributed to the ionization of this top layer.

**Figure 5:**
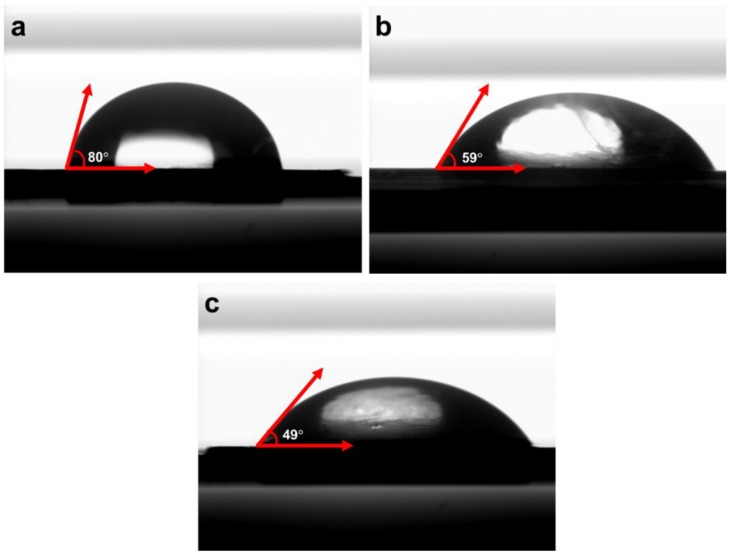
Contact angle measurement of (**a**) pristine PVDF, (**b**) nonwoven/PVDF-PAA and (**c**) nonwoven/PVDF-PAA/GO composite membranes.

The membrane hydrophilicity was investigated by measuring water contact angles on the PVDF, nonwoven/PVDF-PAA and nonwoven/PVDF-PAA/GO composite membranes. The PVDF membrane had a contact angle of 80°. When PAA was blended with PVDF, the top surface had a reduced contact angle of 59° (Figure 5a,b). This confirms the PVDF-PAA layer was more hydrophilic and improves the compatibility with GO layer. When the GO additive was coated on the two-component surface, the contact angle further decreased (49°) due to increasing surface hydrophilicity. This increasing behavior was due to the presence of more C-O, C=O groups in GO (Figure 3b). Zhao et al. used direct blending technique to prepare PVDF/GO composite and the contact angle value was 60.5° [49]. Compared with direct blending, the vacuum filtration coating of GO in composite membrane have lower contact angle due to complete GO coverage on the interlayer surface (Figure 4e).

### 3.4. Permeance and Rejection Performance of Dyes and FA Solutions

**Figure 6:**
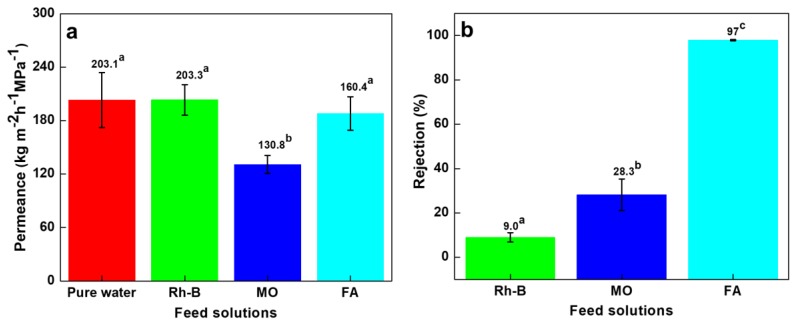
Nonwoven/PVDF-PAA membrane filtration performance: (**a**) permeance and (**b**) rejection data for Rhodamine B, methyl orange (both 10 ppm) and 500 ppm folic acid solutions. Data with different letters are significantly different at 5% significance level. The permeance of pure water is included for comparison.

Dyes (MO and Rh-B) and FA solutions were used as feeds to evaluate the permeance and rejection of composite membranes at steady state (Figure 6a,b). The two-component composite membrane had microporous structure (Figure 4c,d) and demonstrated high pure water permeance of 203.1 kg m^−2^ h^−1^ MPa^−1^ (Figure 6a). The permeance of Rh-B (203.3 kg m^−2^ h^−1^ MPa^−1^, Figure 6a) was not significantly different from the pure water. The Rh-B was nearly un-rejected (at a 9% rejection rate, Figure 6b) probably due to the more neutral charge (with a zeta potential of −8.6 mV in aqueous solution). There was not a significant accumulation of rejected Rh-B molecules at the upstream side of the membrane, therefore the water flux did not deviate from that of the pure water feed.

The permeance was reduced to 130.8 kg m^−2^ h^−1^ MPa^−1^ for MO (Figure 6a), with a rejection rate of 28.3% (Figure 6b). This permeance value was 65% of pure water permeance (which was 203 kg m^−2^ h^−1^ MPa^−1^). The MO molecules has a lower molecular weight than Rh-B (327.33 vs. 479.02 Da, Table 1) but exhibits negative surface charge of −31.6 mV. If we based on sieving mechanism, Rh-B would have been expected to show higher rejection than MO. In contrary, the MO dye was more rejected as compared with Rh-B molecules. This anionic MO dye was rejected on the PVDF-PAA surface probably due to the Donnan exclusion mechanism [47]. The electrostatic and intermolecular interactions between different charged dyes (cationic or anionic) and negatively charged membrane plays an important role for rejection behavior, as pointed out in Ghaffar et al. [33] work.

Similar rejection behavior to MO dye also occurred in FA solution, which molecule owns two carboxylic acid groups. However, a much higher rejection rate (97%, Figure 6b) was obtained for FA. The higher negative surface charge (Table 1) and larger aggregate particle size (1.1 ± 0.2 µm) of FA enhanced the strong repulsion effect and exhibited higher FA rejection rate. The micron-sized FA aggregates were observed in previous work [50] due to strong dimer/oligomer formation and low solubility in water [43,51]. Surprisingly, the FA permeate flux was not sacrificed under the high rejection circumstance. The permeance was 188.1 kg m^−2^ h^−1^ MPa^−1^ for the FA solution, about 93% as the pure water permeance. It is recognized that a reduced permeation flux is typically accompanied with a high rejection for the filtration processes. The rejected species tend to form a concentrated boundary layer or cake layer adjacent to the membrane surface and reduce driving force for filtrate transportation [52]. Comparing the results of MO and FA, the FA had a higher rejection and flux than MO, indicating other factors also affect the flow behavior. As we anticipate that charge repulsion between solute and membrane surface contribute to the degree of solute rejection, the same interaction may loosen the formation of solute cake layer or boundary layer, especially at a very low zeta potential (−45 mV) of the FA solution. Consequently, the FA flux was higher than that of MO.

**Figure 7:**
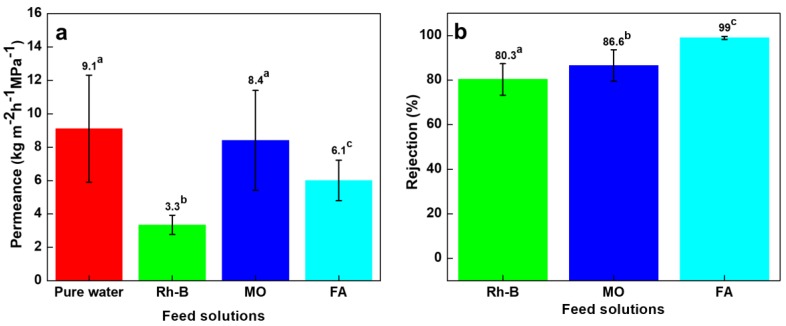
Nonwoven/PVDF-PAA/GO membrane filtration performance: (**a**) permeance and (**b**) rejection values for Rhodamine B, methyl orange (both 10 ppm) and 500 ppm folic acid solutions. Data with different letters are significantly different at 5% significance level. The permeance of pure water is included for comparison.

After GO deposition onto the intermediate substrate (three-component composite), the pure water permeance was significantly declined to 9.1 kg m^−2^ h^−1^ MPa^−1^ (Figure 7a) from the control membrane without GO (with a permeability of 203 kg m^−2^ h^−1^ MPa^−1^ (Figure 6a). Similarly, the permeance of Rh-B solution reduced from 203.3 to 3.3 kg m^−2^ h^−1^ MPa^−1^, while the permeance of MO solution dropped from 130.8 to 8.4 kg m^−2^ h^−1^ MPa^−1^. Moreover, the FA solution permeance value dropped to 6.1 kg m^−2^ h^−1^ MPa^−1^. All the samples showed reduced permeances by 10–15 times after the GO deposition on the composite membrane. The reduction of permeation value was due to that GO formed a dense layer and pore blocking effects on the top surface of the composite membrane. These GO nanosheets are known to form intermolecular hydrogen-bonding and become barrier layer toward water permeation. Similarly, Zhan et al. reported that the GO composite ascribed to the increasing mass transfer resistance, thereby decreased permeance in GO composite membrane than those of pristine sample [4]. In addition, the inner pores, inter-edge spaces and interlayer sieving channels of GO membrane were also responsible for molecular diffusivity and transport pathways [53]. We have estimated that the interlayer spacing for permeation in this GO composite was only a few nanometers [20]. The flow was largely suppressed through this laminated GO layer.

**Figure 8:**
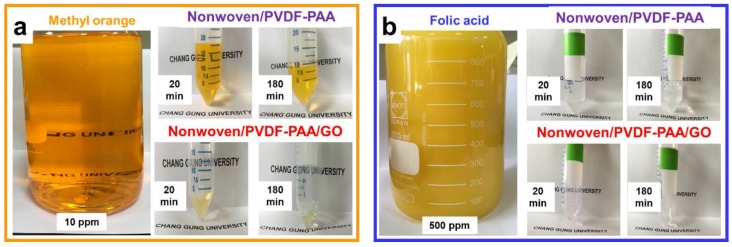
Photographs of (**a**) methyl orange and (**b**) folic acid permeate solutions during various filtration times through nonwoven/PVDF-PAA and nonwoven/PVDF-PAA/GO composite membranes, respectively.

At the same time, such restricted passage in the nonwoven/PVDF-PAA/GO composite membrane benefits solute removal. The rejection efficiency values of Rh-B and MO were dramatically enhanced from 9% to 80.3% and from 28.3% to 86.6%, respectively, as shown in Figure 7b. The rejections of Rh-B and MO dyes were increased 8-folds and 3-folds, as compared to nonwoven/PVDF-PAA composite membrane. This was owing to the uniform nanosized interlayer spacing of the GO film [20], which restricts solute passage. The photographic images of MO solution (Figure 8a) confirm that the nonwoven/PVDF-PAA/GO membrane resulted in much clearer MO filtrate than that without GO deposition (Figure S3), which resulted in yellowish filtrate. In the case of FA, almost complete rejection efficiency (99%) was obtained using the nonwoven/PVDF-PAA/GO composite. This rejection rate was slightly increased (from 97% to 99%) due to the nanosize GO dense layer as compared with nonwoven/PVDF-PAA composite membrane. The photos of the FA filtrates indicated colorless collected fluid (Figure 8b and Figure S4) using either membranes, indicating that both membranes are promising for FA concentration.

### 3.5. Permeance and Rejection Performance of BSA Solution

**Figure 9:**
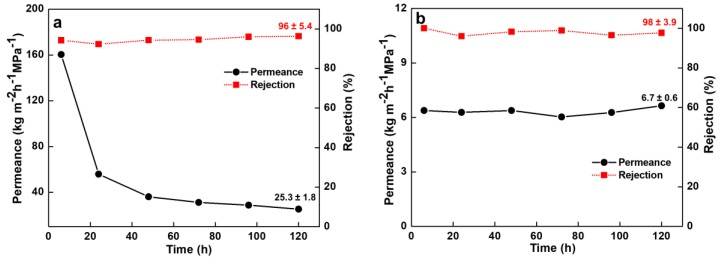
Typical time-dependent permeance and bovine serum albumin solution rejection values through (**a**) nonwoven/PVDF-PAA and (**b**) nonwoven/PVDF-PAA/GO composite membranes.

BSA was selected as the last model molecule to investigate the filtration behavior on the membranes. The nonwoven/PVDF-PAA composite membrane showed stable rejection efficiency (Figure 9a) during 120 h filtration process. The molecular size of the BSA foulant is larger (~200 nm [54,55]) than the pore size of nonwoven/PVDF-PAA composite membrane (75–100 nm). So the BSA seldom penetrated into the pores and only cause surface fouling or adsorption [56]. The BSA was rejected at 96% through the nonwoven/PVDF-PAA composite membrane (Figure 9a). The permeance declined from 160.4 kg m^−2^ h^−1^ MPa^−1^ at initial to 25.3 kg m^−2^ h^−1^ MPa^−1^ at 120 h elapsed time. The permeance decline (from 80% to 13% of pure water permeance) phenomena was due to the concentration polarization or cake formation of the rejected BSA molecules on the upstream surface of the PVDF-PAA layer.

In the case of nonwoven/PVDF-PAA/GO composite membrane, the protein was further rejected at 98% (Figure 9b). The GO dense layer covered the surface porous structure of nonwoven/PVDF-PAA and further blocked the passage of BSA molecules. The interlayer spacing of the laminated GO top layer is several nanometers [20] and can highly reject the larger size BSA molecules. Moreover, the hydrophilicity and negative surface charge of GO (−33 mV) [20] may repel the BSA molecules (which is negatively charged [57]) according to Donnan exclusion theory [58]. The initial permeance value of the protein solution through the GO composite was reduced to 6.4 kg m^−2^ h^−1^ MPa^−1^, similar reduction in order of magnitude of the dyes and FA solutions as compared with those of two-component membrane. The GO resulted in nanochannel and restricted water flow, therefore lowering the filtration flux. However, these nonwoven/PVDF-PAA/GO composite membranes demonstrated a stable permeance value (from 6.4 kg m^−2^ h^−1^ MPa^−1^ to 6.1 kg m^−2^ h^−1^ MPa^−1^) throughout the 120 h filtration process (Figure 9b). This was owing to the loose cake layer and less boundary layer from rejected and accumulated BSA near the negatively charged surface and GO hydrophilic characters at the top layer of membrane. The hydroxyl groups on the GO composite membrane would initially adsorb more aquatic molecules to form a hydrated layer and steric hindrance on the membrane surfaces [59,60]. Obviously the GO composite membranes can improve anti-fouling property [1,61]. The result confirms that the GO composites membrane maintained a more stable permeance and was more resistant to fouling than the nonwoven/PVDF-PAA composite membrane in the BSA filtration process.

The BSA solution may have lower particle size of primary or dimer molecules with <10 nm size [62]. Therefore, the rejections on both membranes did not reach 100%. However, the rejected BSA on upstream side of feed solution tended to be concentrated at the boundary layer. This increased BSA concentration adjacent to the feed side membrane would aggregate further to form larger protein size [55], thus was highly rejected on these two membranes. This section may be divided by subheadings. It should provide a concise and precise description of the experimental results, their interpretation as well as the experimental conclusions that can be drawn.

## 4. Conclusions

The GO nanosheets were synthesized and deposited on porous nonwoven/PVDF-PAA substrate to form three-component composite membrane. The contact angle and zeta potential measurements confirm higher hydrophilicity and lower negative surface charge in the GO membrane than the control nonwoven/PVDF-PAA membrane. Feed solutions of four model molecules were tested on the two composite membranes to study the filtration performance and separation mechanism. For the nonwoven/PVDF-PAA membrane with microporous pores (75–100 nm), cationic Rh-B and anionic MO dye solutions exhibited low rejection rates (9–28%) and relatively high permeance values (100% and 65% of pure water permeance, which was 203 kg m^−2^ h^−1^ MPa^−1^). The FA-formed aggregates and showed high rejection of 97% with high permeance (80% of pure water permeance). The BSA solution was highly rejected (96%) but had a severe flux decline, from 80% to 13% of pure water permeance. The Donnan exclusion and sieving mechanisms govern the solute passage and water permeation behaviors for the smaller (ionic dyes) and larger solutes (FA and BSA), respectively.

For the GO-deposited membrane, the nanochannels between GO interlayer spacing were the main passage route and all model molecules were rejected. Therefore, the solutes were separated based on sieving mechanism. The rejection rates reached 80.3%, 86.6%, 99% and 98% for Rh-B, MO, FA and BSA solutions, respectively. The permeance values were reduced significantly to a few kg m^−2^ h^−1^ MPa^−1^. However, the GO composite membrane had good antifouling property and showed negligible flux decline.

## Figures and Tables

**Table 1:** Molecular structure, pH, particle size and surface charge analysis of Rh-B, MO, FA and BSA molecules.

Samples	Molecular Weight (Da)	Molecular Structure	Concen-Tration (ppm)	pH	Surface Charge (mV)
Rhodamine-B	479.02	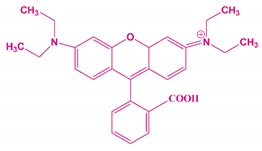	10	4.09	−8.6 ± 1.1
Methyl orange	327.33	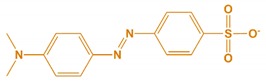	10	5.64	−31.6 ± 1.5
Folic acid	441.4	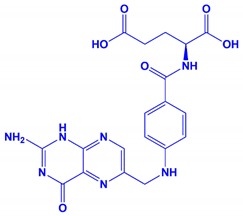	500	3.86	−45 ± 1.1
Bovine serum albumin	66 K	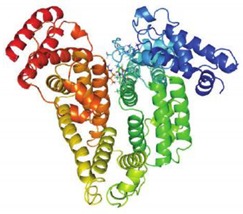	500	6.20	−30 ± 1.0

## Supplementary Materials

The following are available online at https://www.mdpi.com/2079-4991/10/4/792/s1, Figure S1: Fourier transform infrared spectroscopy (FTIR) spectrum of graphite and GO, Figure S2: Raman spectra of graphite and GO, Figure S3: photograph of methyl orange decolorization using nonwoven/PVDF-PAA and nonwoven/PVDF-PAA/GO composite membrane, Figure S4: photograph of folic acid decolorization using nonwoven/PVDF-PAA and nonwoven/PVDF-PAA/GO composite membrane.
