# Supplementary material for: Grafting Thin Layered Graphene Oxide onto the Surface of Nonwoven/PVDF-PAA Composite Membrane for Efficient Dye and Macromolecule Separations
Source: Nanomaterials (Basel). 2020 Apr 20;10(4):792. doi: 10.3390/nano10040792 (PMC7221563; doi:10.3390/nano10040792)
Supplement: Supplementary file 1 [file nanomaterials-10-00792-s001.pdf]

Supplementary Information

Grafting thin layered graphene oxide onto the surface of nonwoven/PVDF-PAA composite membrane for efficient dye and macromolecule separations

Febri Baskoro [1], Selvaraj Rajesh Kumar [1] and Shingjiang Jessie Lue [1,2,3,*]

[1] Department of Chemical and Materials Engineering, Chang Gung University, Guishan District, Taoyuan City 333, Taiwan; febri_baskoro@yahoo.co.id (F.B.); rajeshkumarnst@gmail.com (S.R.K.)

[2] Department of Safety, Health and Environmental Engineering, Ming Chi University of Technology, Taishan District, New Taipei City 243, Taiwan

[3] Department of Orthopedic Surgery, Chang Gung Memorial Hospital, Anle District, Keelung City 204, Taiwan

* Correspondence: jessie@mail.cgu.edu.tw; Tel.: +886-3-2118800 (ext. 5489); Fax: +886-3-2118700

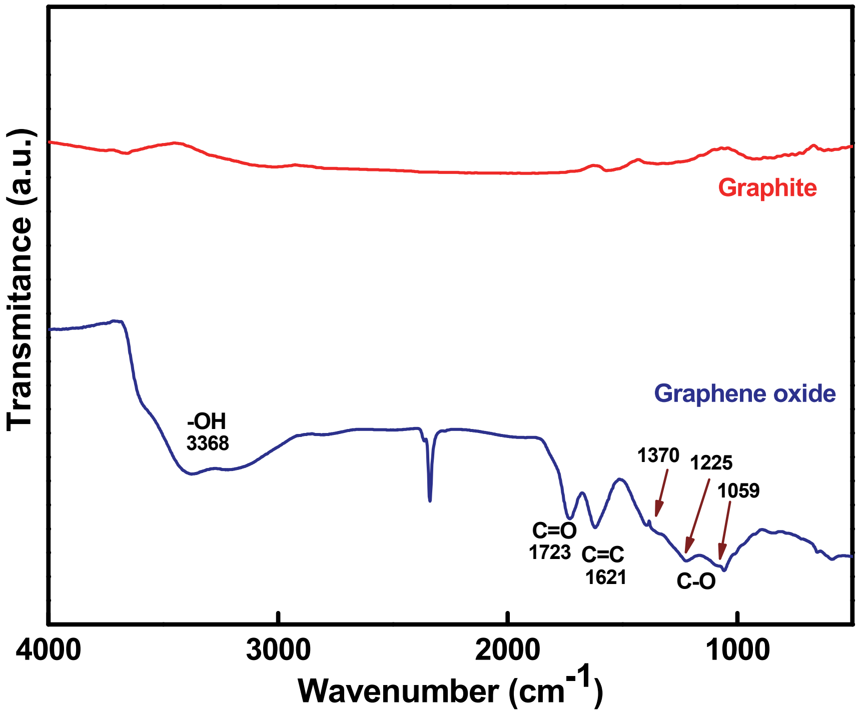

Figure S1. Fourier transform infrared spectroscopy (FTIR) spectrum of graphite and GO.

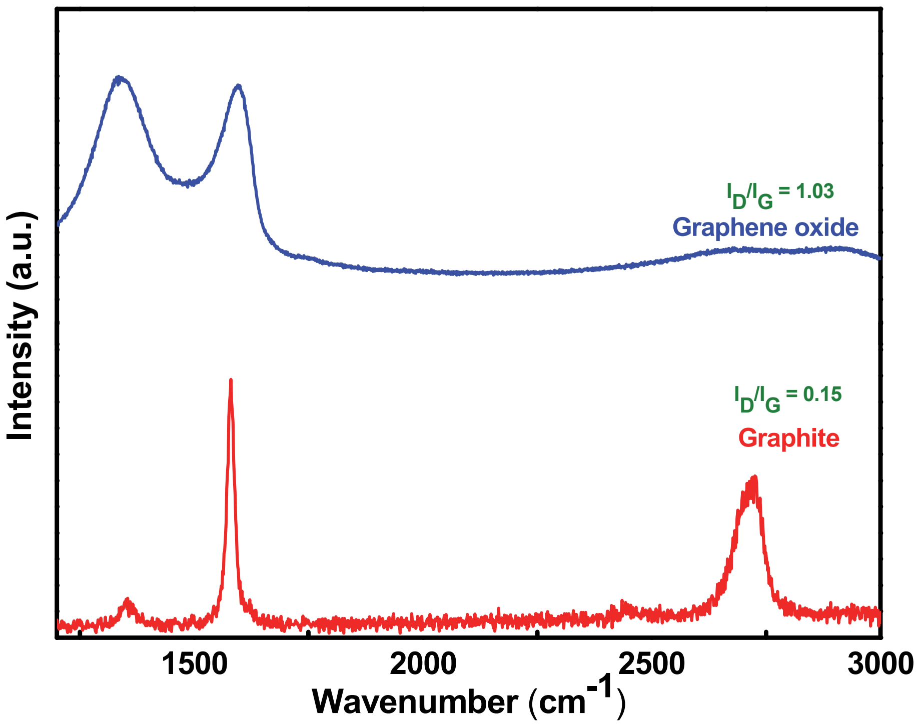

Figure S2. Raman spectrum of graphite and GO.

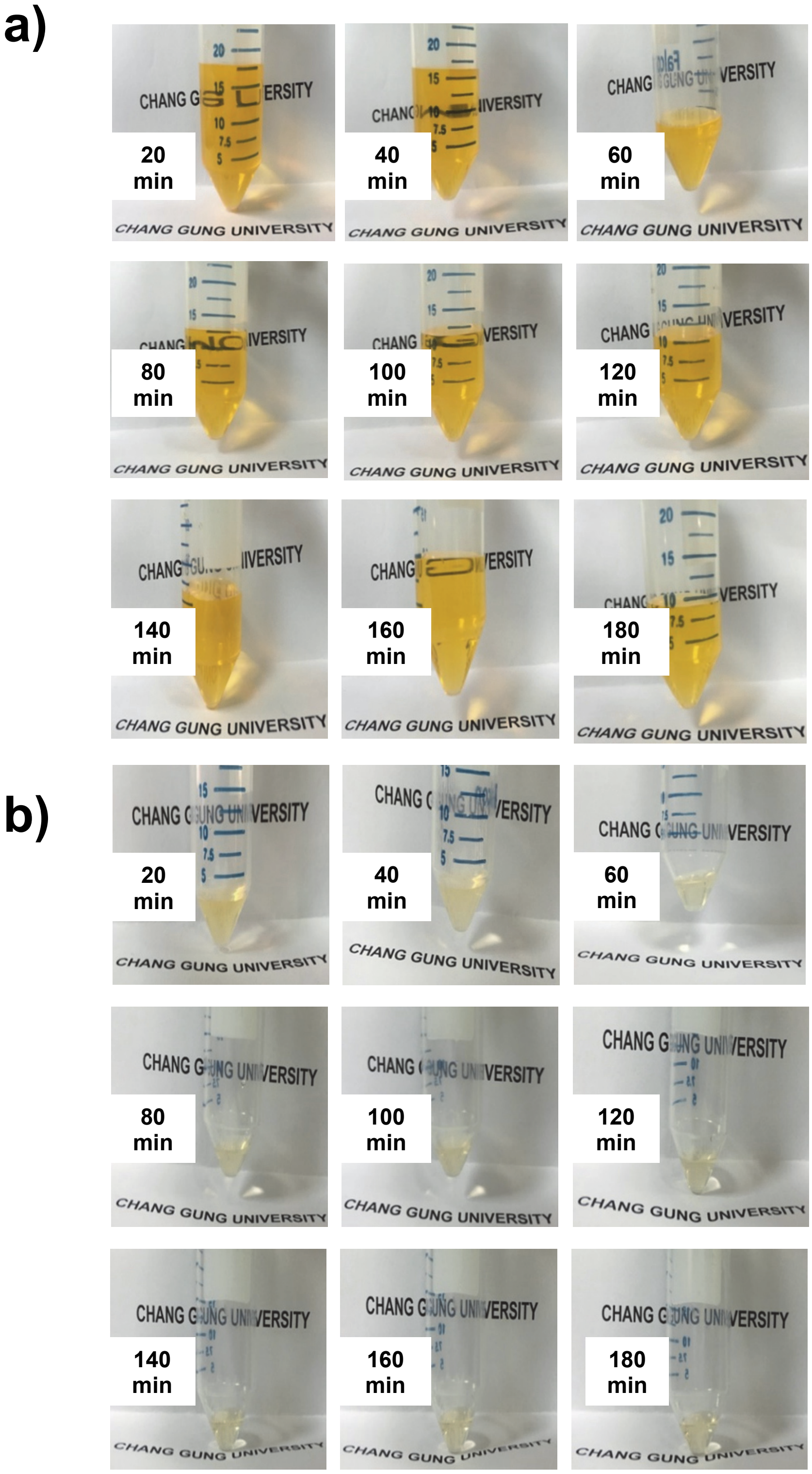

Figure S3. Photograph of methyl orange permeate solutions during various filtration times through (a) nonwoven/PVDF-PAA and (b) nonwoven/PVDF-PAA/GO membranes.

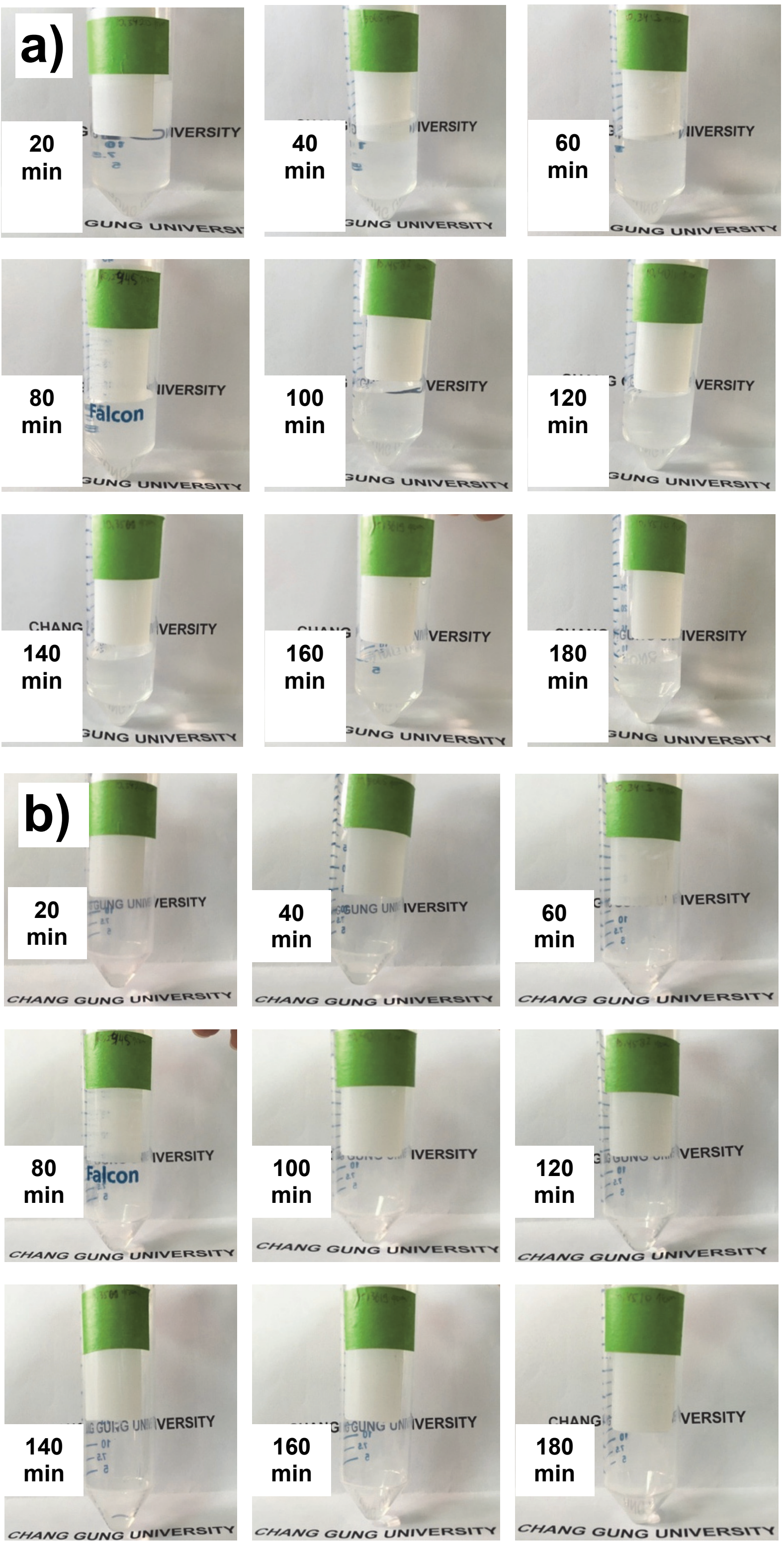

Figure S4. Photograph of folic acid permeate solutions during various filtration times through (a) nonwoven/PVDF-PAA and (b) nonwoven/PVDF-PAA/GO membranes.
